# Development of Prediction Models for Acute Myocardial Infarction at Prehospital Stage with Machine Learning Based on a Nationwide Database

**DOI:** 10.3390/jcdd9120430

**Published:** 2022-12-02

**Authors:** Arom Choi, Min Joung Kim, Ji Min Sung, Sunhee Kim, Jayoung Lee, Heejung Hyun, Hyeon Chang Kim, Ji Hoon Kim, Hyuk-Jae Chang

**Affiliations:** 1 Department of Emergency Medicine, Yonsei University College of Medicine, Seoul 03722, Republic of Korea; 2 Department of Cardiology, Yonsei University College of Medicine, Seoul 03722, Republic of Korea; 3 CONNECT-AI Research Center, Severance Hospital, Yonsei University College of Medicine, Seoul 03722, Republic of Korea; 4 AITRICS, Seoul 06627, Republic of Korea; 5 Department of Preventive Medicine and Public Health, Yonsei University College of Medicine, Seoul 03722, Republic of Korea

**Keywords:** acute myocardial infarction, prediction, machine learning, nationwide prehospital record

## Abstract

Models for predicting acute myocardial infarction (AMI) at the prehospital stage were developed and their efficacy compared, based on variables identified from a nationwide systematic emergency medical service (EMS) registry using conventional statistical methods and machine learning algorithms. Patients in the EMS cardiovascular registry aged >15 years who were transferred from the public EMS to emergency departments in Korea from January 2016 to December 2018 were enrolled. Two datasets were constructed according to the hierarchical structure of the registry. A total of 184,577 patients (Dataset 1) were included in the final analysis. Among them, 72,439 patients (Dataset 2) were suspected to have AMI at prehospital stage. Between the models derived using the conventional logistic regression method, the B-type model incorporated AMI-specific variables from the A-type model and exhibited a superior discriminative ability (*p* = 0.02). The models that used extreme gradient boosting and a multilayer perceptron yielded a higher predictive performance than the conventional logistic regression-based models for analyses that used both datasets. Each machine learning algorithm yielded different classification lists of the 10 most important features. Therefore, prediction models that use nationwide prehospital data and are developed with appropriate structures can improve the identification of patients who require timely AMI management.

## 1. Introduction

Acute myocardial infarction (AMI) is a leading cause of mortality worldwide, despite being one of the known diseases for which standard treatments are well established [1,2]. Early recognition of suspected AMI at the prehospital stage, which is the period between the onset of patients’ symptoms and their arrival at the hospital, and timely management in the hospital, are key factors for improving the survival rate [3,4,5]. The accurate evaluation of risk factors and the likelihood of AMI at the prehospital stage help to provide appropriate prehospital management and rapid transportation to the most appropriate hospital for treatment [6]. 

Predicting AMI and related events during hospitalization using factors derived from hospital data by physicians has been studied extensively and systematically [7,8,9,10,11,12]. 

Conversely, to date, only a few studies have been conducted at the prehospital stage. These studies were performed using only a few variables that influenced the prediction of AMI. Additionally, these efforts and data analyses were not based on organized databases compiled at a national level [13,14,15]. 

Recently, studies have been conducted to predict critical events using machine learning in the field of emergency medicine [16,17,18]. However, all of these predicted critical events are based exclusively on information within the hospital and were conducted based on a single or several centers. Clinical tools using machine learning may be more useful in the pre-hospital stage, where advanced human resources are insufficient, than in hospitals, where physicians with expertise are available. In this context, the Ministry of Science and Information and Communications Technology in Korea has begun a major three-year research project (connected network for emergency medical service (EMS) comprehensive technical-support using artificial intelligence (CONNECT-AI)) to develop an EMS that links information in real time using 5G technology and artificial intelligence spanning all stages of emergency cases [19]. The present study was performed as the initial phase of this project, and it aimed to develop a basic model for critical events at the prehospital stage using machine learning. 

In Korea, paramedics from the National Fire Agency are required to enter detailed information for all transferred patients into the hierarchical structured electrical records in a stepwise manner: EMS run sheets, EMS cardiovascular registry, and AMI-specific records in case of cardiovascular emergencies. Accordingly, we can deduct the decisional process of paramedics to transfer patients to the optimal hospital.

Thus, this study was conducted to (a) develop and present models for predicting AMI at the pre-hospital stage with variables obtained in the two different hierarchical datasets from the nationwide systematic EMS registry, using conventional statistical methods and machine learning algorithms, and (b) compare their performances.

## 2. Materials and Methods

The present study was conducted according to the STROBE and TRIPOD guidelines [20] and was approved by the institutional review boards of Severance Hospital (4-2020-0110). 

### 2.1. Study Design and Setting

This study was a retrospective observational study based on a prospectively collected nationwide dataset from the National Fire Agency and National Emergency Medical Center in Korea. In Korea, the National Fire Agency, which consists of 18 provincial fire departments, oversees the public EMS system. All provincial fire departments operate on a single-tiered and fire-department-based EMS system. The paramedics working at the National Fire Agency comprise level-1 emergency medical technicians (EMTs), level-2 EMTs, and nurses, and are defined according to their qualifications and roles during the transportations of patients from the scene to the hospital. Level-1 EMTs and nurses provide a limited number of advanced treatment techniques, including intravenous fluid administration, such as normal saline and glucose solution; advanced airway placement; injections of specific medications with the supervision of medical directors at the prehospital stage. All patients assessed by the fire-department-based EMS are transported to one of the emergency departments (EDs) for which hospital resources and the distance from the scene are considered optimal. A nationwide fire-department-based EMS quality management program was established in 2011 for major emergency conditions, namely out-of-hospital cardiac arrest, severe trauma, AMI, and acute stroke. With this program, the performance of individual paramedics in each provincial fire department was evaluated, and feedback was provided from the medical director. According to the Rescue and Fire EMS Act, every year, all paramedics at the National Fire Agency are required to receive 40 h of mandatory training in medical skills and knowledge [21]. 

In Korea, EDs are designated at levels 1, 2, or 3 by the Ministry of Health and Welfare. This designation is based on the ED’s human resources, emergency equipment, and availability of medical service and specialists. By law, level-1 and level-2 EDs must be staffed 24 h/day with board-certified emergency physicians [22]. EDs rated at levels 1 and 2 are evaluated annually by the Ministry of Health and Welfare in accordance with the EMS Act to confirm whether they can provide high-level emergency medical care. The designation of levels 1 and 2 can change according to this result. In 2016, 2017, and 2018, the number of sites designated as level-1 EDs and level-2 EDs were 31, 36, and 36, and 120, 119, and 118, respectively [23]. 

### 2.2. Selection of Participants

Patients aged >15 years who were transferred by the fire-department-based EMS to EDs from January 2016 to December 2018 were enrolled. Among them, patients whose EMS cardiovascular registry had been activated were included. Patients whose diagnosis code failed to match data from the National Emergency Department Information System (NEDIS) were excluded.

### 2.3. Data Collection and Processing

The data for the present study were extracted from the following sources: EMS run sheets and the EMS cardiovascular registry, which are managed by the National Fire Agency, and NEDIS, which is operated by the National Emergency medical center in Korea. EMS run sheets are electronically stored to provide a basic EMS operation information repository in the National Fire Agency. The age, gender, past medical history, mental status, and vital signs of the patient at the prehospital stage were extracted from the EMS run sheets. In cases where the symptoms suggested cardiovascular emergencies at the prehospital stage, based on the information recorded on the EMS run sheet, paramedics from the National Fire Agency were required to enter detailed information for AMI screening in the EMS cardiovascular registry. Cardiovascular emergencies are defined as cases in which patients have chest pain, dyspnea, palpitations, syncope, or other suspected cardiovascular events. The EMS cardiovascular registry is a two-step entry system. In the first step, chief complaints, accompanying symptoms, and the onset of symptoms, as well as the location, characteristic, intensity, radiation, duration, and aggravating and relieving factors of chest pain, are evaluated. Based on this information, the paramedics record whether the AMI was presumptive. In cases of suspected AMI, additional information is obtained from the patient and recorded. This additional information includes the following: response of sublingual nitroglycerin (NTG), 3-lead and 12-lead electrocardiographic (ECG) findings, and thrombolysis in myocardial infarction (TIMI) risk score based on the exclusion of cardiac enzyme marker findings. Since 2013, the EMS cardiovascular registry has been amended four times by the quality management committee of experts. The EMS quality management program is in progress, based on data from this registry. 

NEDIS is a computerized system used to collect and analyze the medical information of patients who visit EDs in Korea. This system was developed to evaluate the statuses of the EDs in each medical institution and assess the quality of emergency care. These data include different types of information, such as the emergency care and procedures performed during ED visits, as well as the disposition and diagnosis of each patient at the time of ED discharge. This registry is managed according to the standardized protocol distributed by the National Emergency medical center, has been revised several times up until January 2019, since its establishment in 2003, and has been updated to version 3.2. 

In this study, the information from the EMS cardiovascular registry at the prehospital stage was linked to the diagnosis code of NEDIS to confirm whether the patients had been diagnosed with AMI upon admission to the EDs. The matching variables between the two databases were age, gender, the location of the patient, and the time of arrival to the ED (±10 min).

### 2.4. Model Development

**Figure 1:**
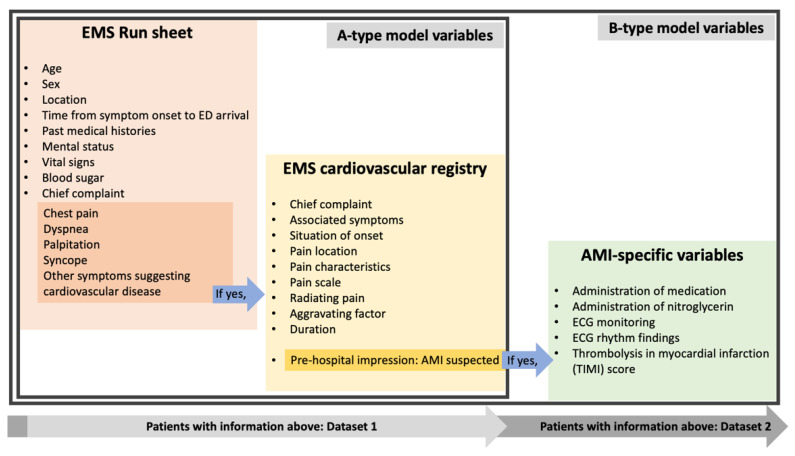
Flow chart for the derivation of datasets (EMS: emergency medical service; ED: emergency department; AMI: acute myocardial infarction; ECG: electrocardiogram).

We developed models to predict AMI diagnosis based on the use of conventional logistic regression and machine learning algorithms. In cases where data were missing, multiple imputations were performed based on fully conditional specifications [24] with SAS (version 9.4, SAS Inc., Cary, NC, USA). The study population was divided into training and testing cohorts, and both subsets were used equally for conventional logistic regression and machine learning analysis. The training and testing cohorts were random samples of 70% and 30% of the entire patient group in the EMS cardiovascular registry, respectively (Dataset 1). The analysis was performed by splitting the training and testing cohorts of the group of patients who were suspected by paramedics to experience an AMI at a ratio of 7:3 (Dataset 2) (Figure 1). 

We derived A-type models with general information from EMS run sheets, and information about cardiovascular symptoms from both datasets from the EMS cardiovascular registry. B-type models were derived using AMI-specific variables from the EMS cardiovascular registry in Dataset 2, such as sublingual NTG administration, ECG findings, and TIMI scores, which were added to the variables in the A-type models (Figure 1).

We developed prediction models using the following five machine learning algorithms: multilayer perceptron (MLP), extreme gradient boosting (XGB), elastic net (EN), random forest, and support vector machine. These algorithms provide calculation probability methods, or functions that map the predictor value to a corresponding probability between zero and one. MLP and XGB were selected for final model derivation, as they yielded the best accuracies between all the algorithms used. MLPs were constructed by stacking layers of perceptrons and were inspired from the neuronal structure of animals. MLPs receive weighted sums of input features and convert them into output signals by using activation functions such as the sigmoid or rectified linear unit. MLPs can effectively capture complicated patterns of data in the form of a nonlinear model when trained with large datasets. The XGB is a gradient-boosted trees model in which gradient boosting is an ensemble method that repeatedly trains new predictors to minimize the residual error of the previous predictor model. As a nonparametric algorithm, XGB is flexible and scalable to various data problems.

### 2.5. Outcome

The primary outcome was a diagnosis of AMI at the time of ED discharge. AMI was defined as the International Classification of Diseases-10 (ICD-10) code I21, which was collected from NEDIS.

### 2.6. Descrimination and Callibration Plot of the Model

The predicted performance was evaluated based on the calculation of the area under the receiver operating characteristic curve (AUC), with 95% confidence intervals for each prediction model, and the performances of the statistical method and machine learning model were compared [25,26]. The predicted performance was also assessed based on sensitivity, specificity, positive predictive value, and negative predictive value. All performance indexes referred to one specific cut-off value, calculated to maximize the Youden index (defined as sensitivity + specificity of 1). The model calibration was assessed by comparing the observed and predicted event probabilities. By plotting two probabilities of event occurrence and conducting the Hosmer–Lemeshow test—a measure of the fit of the model—the observed and predicted risks were compared [27].

### 2.7. Analysis

The continuous variables were analyzed using the independent t-test or Wilcoxon rank-sum test, and the categorical variables were analyzed and compared by the Chi-squared or Fisher’s exact tests. Unadjusted odds ratios with 95% confidence intervals (CIs) for each variable of the study outcome in the training cohort were calculated using the logistic regression analysis. All statistical analyses were conducted with SAS software version 9.4 (SAS Institute, Cary, NC, USA) and R Statistical Package version 3.4.3 (www.R-project.org (accessed on 1 October 2022)). Furthermore, we used TensorFlow version 1.13.1 (www.tensorflow.org (accessed on 1 October 2022)) and scikit-learn version 1.0.2 (www.scikit-learn.org (accessed on 1 October 2022)) libraries in the python (version 3.6.9, www.python.org (accessed on 1 October 2022)) programming environment for machine learning modeling. The statistical significance criterion was set to be two-sided, and *p* values < 0.05 were considered statistically significant.

## 3. Results

### 3.1. Characteristics of Study Subjects

**Figure 2:**
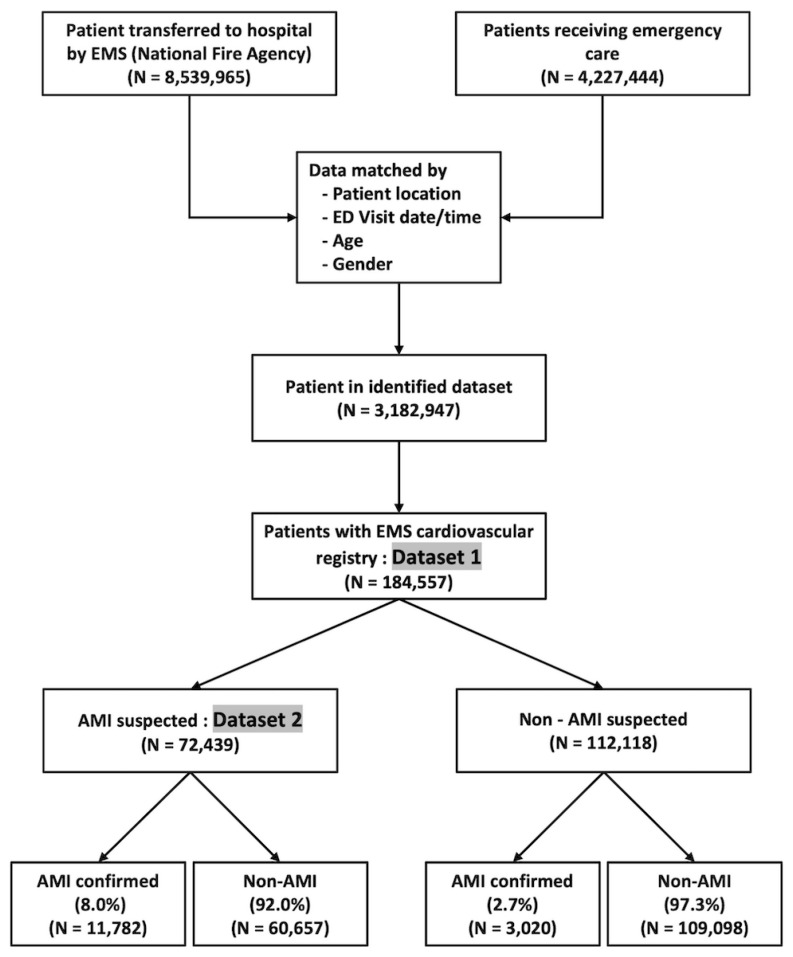
Flow chart of conducted study (EMS: emergency medical service; ED: emergency department; AMI: acute myocardial infarction).

By matching the data of 8,539,965 fire-department-based EMS-transferred patients and 4,227,444 patients who received emergency care in EDs with NEDIS data, 3,182,947 patients were identified as full datasets. Among them, the number of patients in the EMS cardiovascular registry was 184,557. During their transfer to the ED, 72,439 patients were considered to have AMI at the prehospital stage (by paramedics), and 112,118 were considered to have a low probability of AMI. Of the 72,439 patients who were suspected to have suffered AMI, 11,782 (8.0%) patients were confirmed to have AMI at the ED. By contrast, only 2.7% of patients in the patient group that was assigned a low probability for suffering from AMI were diagnosed with AMI (Figure 2). The baseline characteristics of the training and test datasets are shown in Supplementary Table S1. Furthermore, we performed univariate analysis of individual variables from Datasets 1 and 2, and the associations between each variable and outcome were represented as odds ratios. The variables for analysis are shown in Supplementary Table S2.

### 3.2. Main Results

The predictive performances and test characteristics of the models derived using machine learning and conventional statistical analysis are presented in Table 1.

**Figure 3:**
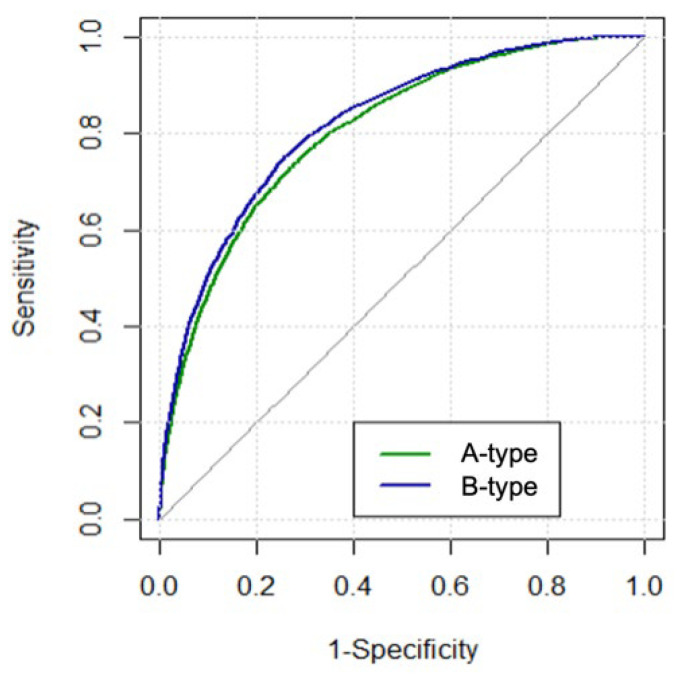
Comparison of discriminative performance between A- and B-type models derived using the conventional logistic regression method.

For models derived using the conventional logistic regression method in Dataset 2, the AUC for the A-type model was 0.808, and the AUC for the B-type model was 0.824. The predictive performance of the B-type models was significantly superior compared with that of the A-type models (*p* = 0.02; Figure 3).

**Figure 4:**
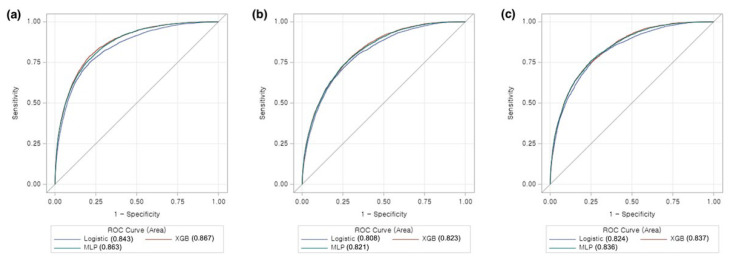
Receiver operating characteristic curves of A-type models with Dataset 1 (**a**) and Dataset 2 (**b**), and B-type models with Dataset 2 (**c**) (ROC: receiver operating characteristic; XGB: Extreme gradient boosting; MLP: Multilayer perceptron).

Figure 4 shows the comparisons of the predictive performances of each model derived from machine learning and the conventional logistic regression model. The A-type models derived from Dataset 1 with XGB and MLP yielded higher predictive performances than the model derived with logistic regression (*p* < 0.01). In this case, the AUCs of XGB and MLP were 0.867 (95% CI: 0.860–0.874) and 0.863 (95% CI: 0.856–0.870), respectively. Likewise, in the A-type models derived from Dataset 2, the AUCs for the XGB- and MLP-derived models were 0.823 (95% CI: 0.815–0.832) and 0.821 (95% CI: 0.812–0.830), respectively. Both models exhibited significantly higher predictive performances than those derived from the conventional logistic regression models (*p* = 0.01 and *p* = 0.03, respectively). In the B-type models, the AUCs for the XGB- and MLP-derived models were 0.837 (95% CI: 0.829–0.846) and 0.836 (95% CI: 0.828–0.845), respectively. The machine learning-derived models also exhibited statistically superior discriminative ability than the logistic regression-derived models (*p* = 0.02 and *p* = 0.03, respectively). 

**Figure 5:**
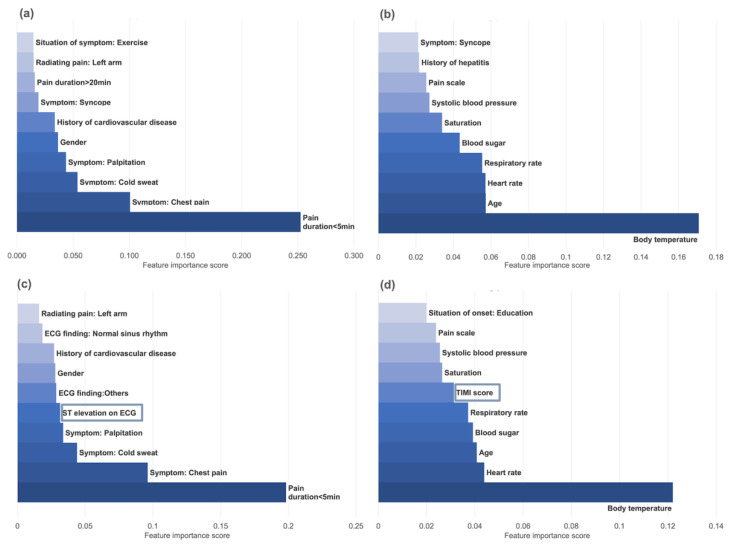
Feature importance score of XGB derived A- (**a**) and B- (**c**) type models compared to MLP- derived A- (**b**) and B- (**d**) type models (XGB: Extreme gradient boosting; MLP: Multilayer perceptron).

For the XGB-derived model, the duration of chest pain, symptoms such as palpitation and cold sweat, gender, and history of cardiovascular disease yielded high-importance scores. In addition, ST segment elevation findings on ECGs yielded high-importance scores in the B-type model. However, for the MLP-derived model, body temperature, age, heart rate, respiratory rate, blood sugar, oxygen saturation, and systolic blood pressure yielded high-importance scores. In addition, TIMI scores yielded high-importance scores in the B-type model. The importance scores of the top 10 features for each machine learning algorithm model are presented in Figure 5. Supplementary Figure S1 shows the calibration plot for all of the tested models. The calibrations were appropriate for all models from both datasets. The Hosmer–Lemeshow plot yielded an insignificant discrepancy between the observed and expected event rates, thus indicating appropriate calibration outcomes for the models. 

## 4. Discussion

The public EMS system of Korea operates with the same authority and policy nationwide; as such, the EMS cardiovascular registry recorded by paramedics has homogeneous characteristics. This was a retrospective study based on nationwide data, and the results confirmed that the EMS cardiovascular registry was efficient in screening patients with cardiovascular risk (predicted to have AMI) at the prehospital stage. In particular, the models that included detailed variables related to cardiovascular risk, such as ECG rhythm analysis, TIMI score, and NTG response, collectively yielded a superior discriminative ability regarding the prediction of AMI. Given that acute coronary syndrome (ACS) is a disease that develops acute and characteristic symptoms, recording the medical history and evaluating the patient’s condition are critical, especially at the prehospital stage. Holmberg et al. [14] highlighted the association between the strength of chest pain and final diagnosis or length of hospital stay. Furthermore, another retrospective study identified the factor that differentiated patients with acute cardiovascular risk among patients without prehospital ECG based on multivariate analysis [15]. However, few previous studies have used multiple variables that influenced the prediction of AMI. Accordingly, the outcomes could not be representative for the entire population, as large-scale data had not been used for analyses. One prospective validation study showed the effectiveness of the history and ECG-only Manchester ACS decision among patients who visited EDs with chest pain. This study revealed that risk stratification tools using non-laboratory variables, including gender, systolic blood pressure, and five historical variables, could exclude ACS in 9.4% of the patients classified in the very-low-risk group [9]. Moreover, one population-based study was conducted to develop a scoring tool to predict sudden cardiac arrest at the prehospital stage based on 8112 STEMI cases. According to this study, factors such as age, diabetes mellitus, obesity, shortness of breath, and the time interval between the development of symptoms and EMS call were associated with 452 sudden cardiac death cases [28]. Decision-making tools that do not require the measurement of biomarkers to assess cardiovascular risk improve risk stratification and are more important in pre-hospital settings. Thus, this study has the significance of developing models that predict AMI through information that is systematically obtained in the pre-hospital stage. In addition, we confirmed that this machine learning-based model had better performance to predict AMI than the conventional method; therefore, it can be a useful tool to support paramedics in making decisions.

In this study, we developed various models for the prediction of AMI at the prehospital stage using machine learning, and verified that XGB and MLP yielded superior performances compared with logistic regression models with conventional statistics. The model that showed the best performance in predicting AMI at the prehospital stage using machine learning algorithms was the XGB model. However, it is not clear why each machine learning method had different discriminant abilities. In addition, previous studies have reported superior predictive performances in the classification or prediction of certain diseases using machine learning; however, the reason for this has not been clarified yet [29,30,31,32].

Given that machine learning is known as a “black box” because of unclear intermediate processes and uncertain interrelationships between variables, machine learning models cannot be mutually applied in terms of diverse feature importance or in approaches for determining risk scores, as these raise concerns in clinical use [33,34]. For this reason, we developed models that could be as descriptive as possible and evaluated the feature importance score for each model. However, it is difficult to compare two models completely one-on-one due to the different directions of interpretation for each model, as well as their different feature importance calculation methods. Thus, each model and its important feature should be interpreted separately and used to emphasize the importance of the variables for model interpretation and verification for application. The features with high-import scores in the XGB-derived models were similar to those of the AMI risk factors calculated with conventional statistical methods, while those that yielded high scores in the MLP-derived models were not.

XGB is a tree ensemble model used for supervised learning, consisting of multiple sets of classification and regression trees. All possible decision-tree structures are added a set at a time, and these sets of trees provide a principled and unified method for optimization of the model. The feature importance of the XGB model in this study was calculated as the result of a relative comparison of the degree of contribution to the classification results for each parameter. In other words, this method is used to calculate the extent of the improvement of the performance when any additional feature is added to the algorithm. This is similar to performance prediction based on conventional logistic regression modeling.

The MLP algorithm is a deep learning algorithm that changes the weight of the input features simultaneously by calculating the sensitivity of changes in the output logit value. It is critical to calculate the feature importance to explain the model because the intermediate processes are unclear and show uncertain interrelationships between parameters, especially in deep learning algorithms. It is assumed that input features with continuous or multi-categorical characteristics that could reflect diversity are more affected, in the case of the MLP algorithm in this study, than features with less variability, such as binary variables. For example, our results indicated that logit values changed in a sensitive manner with respect to changes in body temperature when each feature was compared in a relative manner.

The key differences between the XGB and MLP models are how features are used to develop the model and how the feature importance is calculated. Statistically, it is difficult to determine which one fits better. For example, the AUC for XGB was 0.867 (95% CI 0.860–0.874) and, for MLP, it was 0.863 (95% CI 0.856–0.870) in the A-type model from Dataset 1. Furthermore, each model and its important feature should be interpreted separately and used to emphasize the importance of the variables for model interpretation and verification for application in clinical settings. This result indicates that the types of variables, interactions with other variables, and methods used to calculate their importance can affect the results in each machine learning algorithm. Before machine learning is applied as a clinical tool in the medical field, it should be verified that the features with higher importance scores are suitable for clinical applications.

The method used in the present study setting, based on which prehospital data were collected, proceeded as follows: the paramedics evaluated the patients, identified the patient’s condition and disease severity, initiated the provision of emergency care by examining the patient during transportation to hospital, completed the patient transfer to the appropriate hospital, then recorded the patient data in the EMS registry manually. Data loss is likely to occur because the process depends on memory and on the instantaneous judgment of the paramedics. Furthermore, the computerized system of prehospital evaluation was not configured according to the logical rules of the systematically constructed registry. Therefore, we devoted considerable efforts to data mining. For missing data, we did not perform batch imputation. In other words, we analyzed datasets that were not entered logically and those that should have been recorded but were missed. The assessment and prediction at the prehospital stage should be completed for clinical use, and the emergency physician should be notified before arrival at the ED. In the aforementioned CONNECT AI project, data are collected quickly through automated systems. Patient assessment is then performed with these data at the prehospital stage using the present predictive model, and the results are shared with the hospital in real time. This study suggests that structured and automated systems that handle missing data and errors will increase the clinical usefulness of prehospital records.

The limitations of this study include the following. There is a possibility of bias due to the retrospective design of the study. In particular, the analysis was registry-based rather than being based on the accurate diagnosis from the definite diagnostic method of choice, such as coronary angiography. Second, the NEDIS and EMS registry data could not be merged perfectly, as the exact matching key for both datasets could not be used to ensure the anonymity of the registry data. Third, there was a considerable amount of missing data in the EMS registry owing to the nature of the data based on the retrospective collection method. To solve these issues, the CONNECT-AI project is currently being implemented to prospectively collect prehospital data, using the recognition of voice, video clips of patients, and automatic real-time recordings in ambulances, to predict critical disease by machine learning for further studies. This novel project also aims to ensure consistency and reliability by connecting the database before and after arrival at the hospital. Fourth, the present study focused on the fire-department-based EMS with intermediate service levels in Korea. Therefore, there may be differences in the service levels of EMS in other countries. Thus, caution should be exercised in the generalization of the results of this study.

Lastly, these models have been validated with a retrospective dataset and not in real scenarios. Accordingly, we are planning to conduct a prospective study and validate the usefulness of the developed model to provide guidance for clinical assessment in the prehospital phase.

## 5. Conclusions

This study demonstrates that prediction models that use nationwide prehospital data and are developed with appropriate structures can improve the identification of patients who require timely management. Accurate and rapid prehospital diagnosis and timely treatment determine the clinical course of patients with AMI. The development of a system with advanced technology that can apply our research results in the prehospital practice will be beneficial in improving the clinical outcome of AMI patients.

## Figures and Tables

**Table 1:** Discrimination and test characteristics of acute myocardial infarction prediction models.

Label		Modeling Method	AUROC (95% CI)	Sensitivity (95% CI)	Specificity (95% CI)	Accuracy (95% CI)	PPV (95% CI)	NPV (95% CI)
Dataset 1	A-type model	Logistic	0.843 (0.837, 0.849)	0.750 (0.737, 0.762)	0.785 (0.792, 0.785)	0.785 (0.782, 0.789)	0.241 (0.234, 0.248)	0.972 (0.971, 0.974)
		XGB	0.867 (0.860, 0.874)	0.800 (0.788, 0.812)	0.771 (0.768, 0.775)	0.774 (0.770, 0.778)	0.239 (0.232, 0.245)	0.977 (0.976, 0.979)
		MLP	0.863 (0.856, 0.870)	0.800 (0.788, 0.812)	0.756 (0.752, 0.760)	0.759 (0.756, 0.763)	0.227 (0.220, 0.233)	0.977 (0.975, 0.978)
Dataset 2	A-type model	Logistic	0.808 (0.801, 0.816)	0.721 (0.706, 0.736)	0.743 (0.737, 0.749)	0.739 (0.734, 0.745)	0.355 (0.344, 0.366)	0.931 (0.927, 0.936
		XGB	0.823 (0.815, 0.832)	0.800 (0.787, 0.813)	0.678 (0.672, 0.685)	0.698 (0.692, 0.704)	0.328 (0.318, 0.338)	0.945 (0.941, 0.949)
		MLP	0.821 (0.812, 0.830)	0.800 (0.787, 0.813)	0.673 (0.667, 0.680)	0.694 (0.688, 0.700)	0.324 (0.315, 0.334)	0.945 (0.941, 0.949)
	B-type model	Logistic	0.824 (0.817, 0.831)	0.777 (0.763, 0.791)	0.722 (0.715, 0.728)	0.731 (0.725, 0.737)	0.354 (0.343, 0.364)	0.943 (0.939, 0.947)
		XGB	0.837 (0.829, 0.846)	0.800 (0.787, 0.813)	0.689 (0.682, 0.696)	0.707 (0.701, 0.713)	0.335 (0.325, 0.345)	0.946 (0.942, 0.950)
		MLP	0.836 (0.828, 0.845)	0.800 (0.787, 0.813)	0.700 (0.694, 0.707)	0.717 (0.711, 0.723)	0.344 (0.333, 0.354)	0.947 (0.943, 0.951)

AUROC: area under the receiver operating characteristic curve; CI: confidence interval; PPV: positive predictive value; NPV: negative predictive value; XGB: Extreme gradient boosting; MLP: Multilayer perceptron.

## Data Availability

The data presented in this study are available on request from the corresponding author. The data are not publicly available due to the Personal Information Protection Act in Republic of Korea.

## Supplementary Materials

The following supporting information can be downloaded at: https://www.mdpi.com/article/10.3390/jcdd9120430/s1, Figure S1: the calibration plot for all the tested models; Table S1: Patient demographics for Datasets 1 and 2; Table S2: Odds ratios for event.
