# Supplementary material for: Development of Prediction Models for Acute Myocardial Infarction at Prehospital Stage with Machine Learning Based on a Nationwide Database
Source: J Cardiovasc Dev Dis. 2022 Dec 2;9(12):430. doi: 10.3390/jcdd9120430 (PMC9784963; doi:10.3390/jcdd9120430)
Supplement: Supplementary file 1 [file jcdd-09-00430-s001.zip › jcdd-2007937-supplementary.pdf]

Supplementary Materials

Table S1: Patient demographics for Datasets 1 and 2

Table S1(1): Baseline demographics for the entire cohort of Dataset 1

Variable	Total (N = 184,557)		P value
	**I21.x (Yes = 14,802)**	**I21.x (No = 169,755)**	
Gender (Male)	11,231 (75.87%)	94,750 (55.82%)	<.0001
Age	64.94 ± 13.55	61.25 ± 19.49	<.0001
Location			
Seoul	3,926 (26.52%)	57,398 (33.81%)	<.0001
Others	10,876 (73.48%)	112,357 (66.19%)	
Time from symptom onset to emergency department (ED) arrival (min)	550.98 ± 31,111.94	603.25 ± 35,807.81	0.8634
Hypertension	5,869 (39.65%)	54,664 (32.2%)	<.0001
Diabetes mellitus	3,261 (22.03%)	28,872 (17.01%)	<.0001
Cerebrovascular disease	669 (4.52%)	9,235 (5.44%)	<.0001
Lung disease	499 (3.37%)	18,692 (11.01%)	<.0001
Cardiac disease	4,646 (31.39%)	42,991 (25.33%)	<.0001
Tuberculosis	69 (0.47%)	972 (0.57%)	0.0973
Hepatitis	29 (0.2%)	422 (0.25%)	0.2132
Liver cirrhosis	38 (0.26%)	1,442 (0.85%)	<.0001
Allergy	10 (0.07%)	515 (0.3%)	<.0001
Cancer	429 (2.9%)	12,899 (7.6%)	<.0001
Renal disease	416 (2.81%)	5,617 (3.31%)	0.0011
Others	2,837 (19.17%)	52,558 (30.96%)	<.0001
Mental status			

A: alert	13,867 (93.68%)	153,986 (90.71%)	<.0001
P: react to pain	209 (1.41%)	5,390 (3.18%)	
U: unresponsive	147 (0.99%)	1,855 (1.09%)	
V: react to verbal stimulation	579 (3.91%)	8,524 (5.02%)	
Systolic blood pressure	81.46 ± 20.95	82.19 ± 19.73	<.0001
Diastolic blood pressure	129.81 ± 31.8	131.82 ± 30.19	<.0001
Heart rate	82.79 ± 26.17	94.19 ± 27.53	<.0001
Respiratory rate	19.2 ± 5.19	19.78 ± 5.89	<.0001
Body temperature	36.25±2.46	36.56±2.35	<.0001
Saturation	93.74±11	92.56±11.6	<.0001
Blood sugar	155.58±79.08	151.4±75.15	<.0001
Chief complaint: Chest pain	11,677 (78.89%)	53,846 (31.72%)	<.0001
Chief complaint: Dyspnea	5,474 (36.98%)	93,312 (54.97%)	<.0001
Chief complaint: Palpitation	174 (1.18%)	11,407 (6.72%)	<.0001
Chief complaint: Syncope	334 (2.26%)	29,021 (17.1%)	<.0001
Chief complaint: Others	223 (1.51%)	2,589 (1.53%)	0.8595
Associated symptoms: Nausea	687 (4.64%)	6,989 (4.12%)	0.0022
Associated symptoms: Vomiting	733 (4.95%)	5,423 (3.19%)	<.0001
Associated symptoms: Cold sweat	3,728 (25.19%)	12,743 (7.51%)	<.0001
Associated symptoms: Dizziness	582 (3.93%)	10,448 (6.15%)	<.0001
Associated symptoms: Altered mental status	301 (2.03%)	4,774 (2.81%)	<.0001
Associated symptoms: Others	1,234 (8.34%)	19,360 (11.4%)	<.0001
Situation of onset			
Daily life	9,946 (67.19%)	121,143 (71.36%)	<.0001
Resting/sleeping	2,486 (16.8%)	22,018 (12.97%)	

Working	643 (4.34%)	4,672 (2.75%)	
Sports	462 (3.12%)	1,742 (1.03%)	
On education	6 (0.04%)	369 (0.22%)	
On transfer	536 (3.62%)	7,932 (4.67%)	
On Medical treatment	87 (0.59%)	1,950 (1.15%)	
Others	636 (4.3%)	9,929 (5.85%)	
Pain location: Left	3,656 (24.7%)	17,791 (10.48%)	<.0001
Pain location: Right	745 (5.03%)	4,456 (2.62%)	<.0001
Pain location: Substernal	2,572 (17.38%)	10,013 (5.9%)	<.0001
Pain location: Epigastric	4,212 (28.46%)	18,537 (10.92%)	<.0001
Pain location: Others	1,136 (7.67%)	4,951 (2.92%)	<.0001
Pain characteristics: pressure like	1,707 (11.53%)	7,610 (4.48%)	<.0001
Pain characteristics: tightening/squeezing	4,533 (30.62%)	16,649 (9.81%)	<.0001
Pain characteristics: bursting	558 (3.77%)	1,675 (0.99%)	<.0001
Pain characteristics: dissecting	743 (5.02%)	2,521 (1.49%)	<.0001
Pain characteristics: discomfort	3,320 (22.43%)	18,967 (11.17%)	<.0001
Pain characteristics: Others	1,632 (11.03%)	8,443 (4.97%)	<.0001
Pain scale (NRS)	4.22±3.75	1.4 ± 2.76	<.0001
Radiating pain: left arm	1,477 (9.98%)	3,066 (1.81%)	<.0001
Radiating pain: right arm	610 (4.12%)	1,488 (0.88%)	<.0001
Radiating pain: back	1,021 (6.9%)	4,386 (2.58%)	<.0001
Radiating pain: neck	658 (4.45%)	2,365 (1.39%)	<.0001

Radiating pain: others	518 (3.5%)	2,228 (1.31%)	<.0001
Aggravating factor			
None	10,108 (86.6%)	47,246 (87.74%)	<.0001
Exercise	1,338 (11.46%)	4,754 (8.83%)	
Others	231 (1.98%)	1,846 (3.43%)	
Relieving factor			
None	8,628 (73.89%)	37,204 (69.09%)	<.0001
Nitroglycerin	1,571 (13.45%)	7,607 (14.13%)	
Resting	1,212 (10.38%)	7,380 (13.71%)	
Others	266 (2.28%)	1,655 (3.07%)	
Duration			
Under 5 min	1,294 (11.08%)	9,378 (17.42%)	<.0001
5 to 20 min	2,987 (25.58%)	14,079 (26.15%)	
Over 20 min	7,396 (63.34%)	30,389 (56.44%)	

Table S1(2): Baseline demographics for the training cohort of Dataset 1

Variable	Training set (N = 129,210)		P value
	I21.x (Yes = 10,252)	I21.x (No = 118,958)	
Gender (Male)	7,768 (75.77%)	66,565 (55.96%)	<.0001
Age	64.98 ± 13.54	61.22 ± 19.51	<.0001
Location			
Seoul	2,733 (26.66%)	40,195 (33.79%)	<.0001
Others	7,519 (73.34%)	78,763 (66.21%)	
Time from symptom onset to ED arrival (min)	307.65 ± 7,965.51	593.89 ± 32,228.98	0.0191
Hypertension	4,064 (39.64%)	38,328 (32.22%)	<.0001

Diabetes mellitus	2,252 (21.97%)	20,256 (17.03%)	<.0001
Cerebrovascular disease	449 (4.38%)	6,441 (5.41%)	<.0001
Lung disease	352 (3.43%)	13,115 (11.02%)	<.0001
Cardiac disease	3,208(31.29%)	30,096 (25.3%)	<.0001
Tuberculosis	43 (0.42%)	685 (0.58%)	0.0424
Hepatitis	25 (0.24%)	291 (0.24%)	0.9879
Liver cirrhosis	28 (0.27%)	978(0.82%)	<.0001
Allergy	5 (0.05%)	372 (0.31%)	<.0001
Cancer	304 (2.97%)	9,112 (7.66%)	<.0001
Renal disease	285 (2.78%)	3,897 (3.28%)	0.0065
Others	1,968 (19.2%)	36,860 (30.99%)	<.0001
Mental status			
A: alert	9,606 (93.7%)	107,871 (90.68%)	<.0001
P: react to pain	148 (1.44%)	3,790 (3.19%)	
U: unresponsive	104 (1.01%)	1,287 (1.08%)	
V: react to verbal stimulation	394 (3.84%)	6,010 (5.05%)	
Systolic blood pressure	81.43±20.91	82.16 ± 19.75	0.0006
Diastolic blood pressure	129.65 ± 31.66	131.83 ± 30.23	<.0001
Heart rate	82.92 ± 26.05	94.26 ± 27.57	<.0001
Respiratory rate	19.24 ± 5.28	19.78 ± 5.87	<.0001
Body temperature	36.27 ± 2.29	36.56 ± 2.36	<.0001
Saturation	93.73 ± 11.01	92.55 ± 11.6	<.0001
Blood sugar	155.03 ± 79.21	151.3 ± 75.36	<.0001
Chief complaint: Chest pain	8,063 (78.65%)	37,824 (31.8%)	<.0001
Chief complaint: Dyspnea	3,775 (36.82%)	65,314 (54.91%)	<.0001
Chief complaint: Palpitation	117 (1.14%)	7,983 (6.71%)	<.0001
Chief complaint: Syncope	235 (2.29%)	20,358 (17.11%)	<.0001
Chief complaint: Others	160 (1.56%)	1,762 (1.48%)	0.5236
Associated symptoms: Nausea	481 (4.69%)	4,845 (4.07%)	0.0025
Associated symptoms: Vomiting	519 (5.06%)	3,793 (3.19%)	<.0001
Associated symptoms: Cold sweat	2,587 (25.23%)	8,965 (7.54%)	<.0001
Associated symptoms: Dizziness	402 (3.92%)	7,284 (6.12%)	<.0001
Associated symptoms: Altered mental status	205 (2%)	3,348 (2.81%)	<.0001

Associated symptoms: Others	858 (8.37%)	13,544 (11.39%)	<.0001
Situation of onset			
Daily life	6,892 (67.23%)	84,813 (71.3%)	<.0001
Resting/sleeping	1,719 (16.77%)	15,464 (13%)	
Working	441 (4.3%)	3,294 (2.77%)	
Sports	318 (3.1%)	1,195 (1%)	
On education	4 (0.04%)	270 (0.23%)	
On transfer	376 (3.67%)	5,531 (4.65%)	
On Medical treatment	60 (0.59%)	1,349 (1.13%)	
Others	442 (4.31%)	7,042 (5.92%)	
Pain location: Left	2,542 (24.8%)	12,604 (10.6%)	<.0001
Pain location: Right	529 (5.16%)	3,157 (2.65%)	<.0001
Pain location: Substernal	1,772 (17.28%)	7,037 (5.92%)	<.0001
Pain location: Epigastric	2,894 (28.23%)	12,913 (10.86%)	<.0001
Pain location: Others	787 (7.68%)	3,447 (2.9%)	<.0001
Pain characteristics: pressure like	1,181 (11.52%)	5,312 (4.47%)	<.0001
Pain characteristics: tightening/squeezing	3,175 (30.97%)	11,725 (9.86%)	<.0001
Pain characteristics: bursting	391 (3.81%)	1,178 (0.99%)	<.0001
Pain characteristics: dissecting	532 (5.19%)	1,741 (1.46%)	<.0001
Pain characteristics: discomfort	2,262 (22.06%)	13,358 (11.23%)	<.0001
Pain characteristics: Others	1,092 (10.65%)	5,923 (4.98%)	<.0001
Pain scale(NRS)	4.21 ± 3.75	1.4 ± 2.76	<.0001
Radiating pain: left arm	1,031 (10.06%)	2,118 (1.78%)	<.0001
Radiating pain: right arm	424 (4.14%)	1,050 (0.88%)	<.0001
Radiating pain: back	718 (7%)	3,085 (2.59%)	<.0001
Radiating pain: neck	450 (4.39%)	1,652 (1.39%)	<.0001
Radiating pain: others	362 (3.53%)	1,601 (1.35%)	<.0001
Aggravating factor			
None	6,971 (86.46%)	33,168 (87.69%)	<.0001
Exercise	924 (11.46%)	3,372 (8.91%)	
Others	168 (2.08%)	1,284 (3.39%)	
Relieving factor			
None	5,950 (73.79%)	26,083 (68.96%)	<.0001

Nitroglycerin	1,097 (13.61%)	5,369 (14.19%)	
Resting	824 (10.44%)	5,202 (13.75%)	
Others	174 (2.16%)	1,170 (3.09%)	
Duration			
Under 5 min	916 (11.36%)	6,559 (17.34%)	<.0001
5 to 20 min	2,072 (25.7%)	9,946 (26.3%)	
Over 20 min	5,075 (62.94%)	21,319 (56.36%)	

Table S1(3): Baseline demographics for the test cohort of Dataset 1

**Variable**	**Testing set (N = 55,347)**		**P value**
	**I21.x (Yes = 4,550)**	**I21.x (No = 50,797)**	
Gender (Male)	3,463 (76.11%)	28,185 (55.49%)	<.0001
Age	64.87 ± 13.58	61.32 ± 19.44	<.0001
Location			
Seoul	1,193 (26.22%)	17,203 (33.87%)	<.0001
Others	3,357 (73.78%)	33,594 (66.13%)	
Time from symptom onset to ED arrival (min)	1,099.24 ± 54,826.96	625.17 ± 43,040.07	0.5702
Hypertension	1,805 (39.67%)	16,336 (32.16%)	<.0001
Diabetes mellitus	1,009 (22.18%)	8,616 (16.96%)	<.0001
Cerebrovascular disease	220 (4.84%)	2,794 (5.5%)	0.0582
Lung disease	147 (3.23%)	5,577 (10.98%)	<.0001
Cardiac disease	1,438 (31.6%)	12,895 (25.39%)	<.0001
Tuberculosis	26 (0.57%)	287 (0.56%)	0.9558
Hepatitis	4 (0.09%)	131 (0.26%)	0.026
Liver cirrhosis	10 (0.22%)	464 (0.91%)	<.0001
Allergy	5 (0.11%)	143 (0.28%)	0.0317
Cancer	125 (2.75%)	3,787 (7.46%)	<.0001

Renal disease	131 (2.88%)	1,720 (3.39%)	0.0685
Others	869 (19.1%)	15,698 (30.9%)	<.0001
Mental status			
A: alert	4,261 (93.65%)	46,115 (90.78%)	<.0001
P: react to pain	61 (1.34%)	1,600 (3.15%)	
U: unresponsive	43 (0.95%)	568 (1.12%)	
V: react to verbal stimulation	185 (4.07%)	2,514 (4.95%)	
Systolic blood pressure	81.51 ± 21.04	82.24 ± 19.7	0.0253
Diastolic blood pressure	130.16 ± 32.12	131.79 ± 30.09	0.001
Heart rate	82.5 ± 26.45	94.02 ± 27.45	<.0001
Respiratory rate	19.12 ± 4.97	19.79 ± 5.93	<.0001
Body temperature	36.2 ± 2.81	36.56 ± 2.35	<.0001
Saturation	93.76 ± 10.99	92.6 ± 11.61	<.0001
Blood sugar	156.82 ± 78.8	151.62 ± 74.68	<.0001
Chief complaint: Chest pain	3,614 (79.43%)	16,022 (31.54%)	<.0001
Chief complaint: Dyspnea	1,699 (37.34%)	27,998 (55.12%)	<.0001
Chief complaint: Palpitation	57 (1.25%)	3,424 (6.74%)	<.0001
Chief complaint: Syncope	99 (2.18%)	8,663 (17.05%)	<.0001
Chief complaint: Others	63 (1.38%)	827 (1.63%)	0.2111
Associated symptoms: Nausea	206 (4.53%)	2,144 (4.22%)	0.3256
Associated symptoms: Vomiting	214 (4.7%)	1,630 (3.21%)	<.0001
Associated symptoms: Cold sweat	1,141 (25.08%)	3,778 (7.44%)	<.0001
Associated symptoms: Dizziness	180 (3.96%)	3,164 (6.23%)	<.0001
Associated symptoms: Altered mental status	96 (2.11%)	1,426 (2.81%)	0.0059
Associated symptoms: Others	376 (8.26%)	5,816 (11.45%)	<.0001
Situation of onset			
Daily life	3,054 (67.12%)	36,330 (71.52%)	<.0001

Resting/sleeping	767 (16.86%)	6,554 (12.9%)	
Working	202 (4.44%)	1,378 (2.71%)	
Sports	144 (3.16%)	547 (1.08%)	
On education	2 (0.04%)	99 (0.19%)	
On transfer	160 (3.52%)	2,401 (4.73%)	
On Medical treatment	27 (0.59%)	601 (1.18%)	
Others	194 (4.26%)	2,887 (5.68%)	
Pain location: Left	1,114 (24.48%)	5,187 (10.21%)	<.0001
Pain location: Right	216 (4.75%)	1,299 (2.56%)	<.0001
Pain location: Substernal	800 (17.58%)	2,976 (5.86%)	<.0001
Pain location: Epigastric	1,318 (28.97%)	5,624 (11.07%)	<.0001
Pain location: Others	349 (7.67%)	1,504 (2.96%)	<.0001
Pain characteristics: pressure like	526 (11.56%)	2,298 (4.52%)	<.0001
Pain characteristics: tightening/squeezing	1,358 (29.85%)	4,924 (9.69%)	<.0001
Pain characteristics: bursting	167 (3.67%)	497 (0.98%)	<.0001
Pain characteristics: dissecting	211 (4.64%)	780 (1.54%)	<.0001
Pain characteristics: discomfort	1,058 (23.25%)	5,609 (11.04%)	<.0001
Pain characteristics: Others	540 (11.87%)	2,520 (4.96%)	<.0001
Pain scale (NRS)	4.23 ± 3.75	1.4 ± 2.77	<.0001
Radiating pain: left arm	446 (9.8%)	948 (1.87%)	<.0001
Radiating pain: right arm	186 (4.09%)	438 (0.86%)	<.0001
Radiating pain: back	303 (6.66%)	1,301 (2.56%)	<.0001
Radiating pain: neck	208 (4.57%)	713 (1.4%)	<.0001
Radiating pain: others	156 (3.43%)	627 (1.23%)	<.0001
Aggravating factor			
None	3,137 (86.8%)	14,078 (87.87%)	<.0001
Exercise	414 (11.46%)	1,382 (8.63%)	

Others	63 (1.74%)	562 (3.51%)	
Relieving factor			
None	2,678 (74.1%)	11,121 (69.41%)	<.0001
Nitroglycerin	474 (13.12%)	2,238 (13.97%)	
Resting	370 (10.24%)	2,178 (13.59%)	
Others	92 (2.55%)	485 (3.03%)	
Duration			
Under 5 min	378 (10.46%)	2,819 (17.59%)	<.0001
5 to 20 min	915 (25.32%)	4,133 (25.8%)	
Over 20 min	2,321 (64.22%)	9,070 (56.61%)	

Table S1(4): Baseline demographics for the entire cohort of Dataset 2

**Variable**	**Total (N = 72,439)**		**P value**
	**I21.x (Yes = 11,782)**	**I21.x (No = 60,657)**	
Gender (Male)	9,334 (79.22%)	35,054 (57.79%)	<.0001
Age	63.23 ± 12.95	60.82 ± 17.21	<.0001
Location			
Seoul	2,998 (25.45%)	18,061 (29.78%)	<.0001
Others	8,784 (74.55%)	42,596 (70.22%)	
Time from symptom onset to emergency department (ED) arrival (min)	278.7 ± 7,508.23	498.91 ± 39,102.63	0.2035
Hypertension	4,562 (38.72%)	22,153 (36.52%)	<.0001
Diabetes mellitus	2,374 (20.15%)	10,640 (17.54%)	<.0001
Cerebrovascular disease	443 (3.76%)	2,477 (4.08%)	0.1022
Lung disease	156 (1.32%)	1,351 (2.23%)	<.0001
Cardiac disease	4,038 (34.27%)	30,170 (49.74%)	<.0001
Tuberculosis	54 (0.46%)	306 (0.5%)	0.5145
Hepatitis	18 (0.15%)	136 (0.22%)	0.1234

Liver cirrhosis	23 (0.2%)	277 (0.46%)	<.0001
Allergy	8 (0.07%)	49 (0.08%)	0.6482
Cancer	252 (2.14%)	1,927 (3.18%)	<.0001
Renal disease	241 (2.05%)	1,598 (2.63%)	0.0002
Others	1,984 (16.84%)	13,066 (21.54%)	<.0001
Mental status			
A: alert	11,230 (95.31%)	57,856 (95.38%)	<.0001
P: react to pain	86 (0.73%)	714 (1.18%)	
U: unresponsive	94 (0.8%)	515 (0.85%)	
V: react to verbal stimulation	372 (3.16%)	1,572 (2.59%)	
Systolic blood pressure	80.94±20.86	82.49±19.68	<.0001
Diastolic blood pressure	128.6±31.49	132.39±30.12	<.0001
Heart rate	79.54±25.11	92.57±32.83	<.0001
Respiratory rate	18.79±4.91	18.67±5.08	0.0196
Body temperature	36.23±2.34	36.44±2.32	<.0001
Saturation	95.29±9.59	95.52±9.24	0.0177
Blood sugar	152.43±77.31	148.87±74.15	<.0001
Chief complaint: Chest pain	10,723 (91.01%)	39,795 (65.61%)	<.0001
Chief complaint: Dyspnea	3,331 (28.27%)	19,558 (32.24%)	<.0001
Chief complaint: Palpitation	158 (1.34%)	8,187 (13.5%)	<.0001
Chief complaint: Syncope	188 (1.6%)	5,486 (9.04%)	<.0001
Chief complaint: Others	202 (1.71%)	1,476 (2.43%)	<.0001
Associated symptoms: Nausea	596 (5.06%)	2,856 (4.71%)	0.1026
Associated symptoms: Vomiting	634 (5.38%)	1,944 (3.2%)	<.0001
Associated symptoms: Cold sweat	3,403 (28.88%)	6,908 (11.39%)	<.0001
Associated symptoms: Dizziness	508 (4.31%)	4,343 (7.16%)	<.0001
Associated symptoms: Altered mental status	181 (1.54%)	1,104 (1.82%)	0.0327
Associated symptoms: Others	937 (7.95%)	6,208 (10.23%)	<.0001
Situation of onset			
Daily life	7,740 (65.69%)	42,166 (69.52%)	<.0001
Resting/sleeping	1,982 (16.82%)	9,278 (15.3%)	
Working	592 (5.02%)	1,930 (3.18%)	
Sports	472 (3.62%)	818 (1.35%)	
On education	6 (0.05%)	95 (0.16%)	

On transfer	467 (3.96%)	2,829 (4.66%)	
On medical treatment	54 (0.46%)	428 (0.71%)	
Others	514 (4.36%)	3,113 (5.13%)	
Pain location: Left	3,417 (29%)	13,946 (22.99%)	<.0001
Pain location: Right	668 (5.67%)	2,880 (4.75%)	<.0001
Pain location: Substernal	2,414 (20.49%)	7,565 (12.47%)	<.0001
Pain location: Epigastric	3,827 (32.48%)	13,646 (22.5%)	<.0001
Pain location: Others	1,014 (8.61%)	3,382 (5.58%)	<.0001
Pain characteristics: pressure like	1,595 (13.54%)	5,889 (9.71%)	<.0001
Pain characteristics: tightening/squeezing	4,294 (36.45%)	13,637 (22.48%)	<.0001
Pain characteristics: bursting	530 (4.5%)	1,334 (2.2%)	<.0001
Pain characteristics: dissecting	693 (5.88%)	1,866 (3.08%)	<.0001
Pain characteristics: discomfort	2,973 (25.23%)	13,421 (22.13%)	<.0001
Pain characteristics: Others	1,446 (12.27%)	5,605 (9.24%)	<.0001
Pain scale (NRS)	4.98±3.62	3.08 ± 3.45	<.0001
Radiating pain: left arm	1,421 (12.06%)	2,710 (4.47%)	<.0001
Radiating pain: right arm	592 (5.02%)	1,271 (2.1%)	<.0001
Radiating pain: back	973 (8.26%)	3,592 (5.92%)	<.0001
Radiating pain: neck	633 (5.37%)	2,005 (3.31%)	<.0001
Radiating pain: others	493 (4.18%)	1,814 (2.99%)	<.0001
Aggravating factor			
None	9,227 (86.05%)	34,791 (87.43%)	<.0001
Exercise	1,282 (11.96%)	3,875 (9.74%)	
Others	214 (2%)	1,129 (2.84%)	
Relieving factor			
None	7,831 (73.03%)	25,718 (64.63%)	<.0001
Nitroglycerin	1,550 (14.45%)	7,347 (18.46%)	
Resting	1,109 (10.34%)	5,584 (14.03%)	
Others	233 (2.17%)	1,146 (2.88%)	
Duration			
Under 5 min	1,120 (10.44%)	6,533 (16.42%)	<.0001

5 to 20 min	2,758 (25.72%)	10,851 (27.27%)	
Over 20 min	6,845 (63.83%)	22,411 (56.32%)	
Administration of medication	1,832 (16.61%)	9,957 (18.11%)	<.0001
Administration of nitroglycerin	2,203 (18.7%)	6,160 (10.16%)	<.0001
ECG monitoring device	11,207 (95.12%)	56,743 (93.55%)	<.0001
Electrode	9,973 (88.99%)	51,309 (90.42%)	<.0001
AED (automated external defibrillator)	1,234 (11.01%)	5,434 (9.58%)	
ECG rhythm findings			
Normal sinus rhythm	4,951 (44.18%)	30,960 (54.56%)	<.0001
Sinus tachycardia	744 (6.64%)	8,975 (15.82%)	
Sinus bradycardia	1,030 (9.19%)	2,756 (4.86%)	
Narrow QRS tachycardia	265 (2.36%)	2,419 (4.26%)	
Wide QRS tachycardia	204 (1.82%)	613 (1.08%)	
Atrioventricular (AV) block	112 (1%)	380 (0.67%)	
Other AV blocks	38 (0.34%)	141 (0.25%)	
Unknown	1,033 (9.22%)	3,758 (6.62%)	
Others	2,830 (25.25%)	6,741 (11.88%)	
AV block			
Second degree AV block	333 (45.93%)	2,159 (48.68%)	<.0001
Third degree AV block	229 (31.59%)	1,358 (30.62%)	
Other AV block	163 (22.48%)	918 (20.7%)	
Wide QRS tachycardia			
Regular	155 (75.98%)	470 (76.67%)	<.0001
Irregular	49 (24.02%)	143 (23.33%)	
Narrow QRS tachycardia			
Regular	173 (65.28%)	1,682 (69.53%)	0.0005
Irregular	92 (34.72%)	737 (30.47%)	
ST segment elevation	3,479 (29.53%)	9,102 (15.01%)	<.0001

Thrombolysis in myocardial infarction (TIMI) score	1.56 ± 1.2	1.15 ± 1.15	<.0001

Table S1(5): Baseline demographics for the training cohort of Dataset 2

**Variable**	**Training set (N = 50,713)**		**P value**
	**I21.x (Yes = 8,222)**	**I21.x (No = 42,491)**	
Gender (Male)	6,515 (79.24%)	24,629 (57.96%)	<.0001
Age	63.26 ± 13.02	60.83 ± 17.2	<.0001
Location			
Seoul	2,076 (25.25%)	12,714 (29.92%)	<.0001
Others	6,146 (74.75%)	29,777 (70.08%)	
Time from symptom onset to ED arrival (min)	176.67 ± 4,375.99	575.82 ± 46,519.7	0.0837
Hypertension	3,189 (38.79%)	15,485 (36.44%)	<.0001
Diabetes mellitus	1,651 (20.08%)	7,441 (17.51%)	<.0001
Cerebrovascular disease	329 (4%)	1,742 (4.1%)	0.6804
Lung disease	108 (1.31%)	935 (2.2%)	<.0001
Cardiac disease	2,804 (34.1%)	21,136 (49.74%)	<.0001
Tuberculosis	38 (0.46%)	232 (0.55%)	0.339
Hepatitis	11 (0.13%)	95 (0.22%)	0.1027
Liver cirrhosis	13 (0.16%)	201 (0.47%)	<.0001
Allergy	5 (0.06%)	33 (0.08%)	0.6093
Cancer	187 (2.27%)	1,356 (3.19%)	<.0001
Renal disease	167 (2.03%)	1,105 (2.6%)	0.0025
Others	1,419 (17.26%)	9,146 (21.52%)	<.0001
Mental status			
A: alert	7,860 (95.6%)	40,532 (95.39%)	0.0007
P: react to pain	64 (0.78%)	506 (1.19%)	
U: unresponsive	56 (0.68%)	366 (0.86%)	
V: react to verbal stimulation	242 (2.94%)	1,087 (2.56%)	
Systolic blood pressure	81.07±20.73	82.45±19.68	<.0001
Diastolic blood pressure	128.91±31.24	132.26±30.14	<.0001
Heart rate	80.01±25.17	92.52±32.85	<.0001

Respiratory rate	18.79±4.71	18.65±5.04	0.0122
Body temperature	36.24±2.27	36.43±2.34	<.0001
Saturation	95.32±9.51	95.54±9.21	0.0464
Blood sugar	152.11±77.4	148.66±74.06	0.0002
Chief complaint: Chest pain	7,469 (90.84%)	27,945 (65.77%)	<.0001
Chief complaint: Dyspnea	2,335 (28.4%)	13,589 (31.98%)	<.0001
Chief complaint: Palpitation	107 (1.3%)	5,648 (13.29%)	<.0001
Chief complaint: Syncope	134 (1.63%)	3,880 (9.13%)	<.0001
Chief complaint: Others	143 (1.74%)	1,044 (2.46%)	<.0001
Associated symptoms: Nausea	413 (5.02%)	1,990 (4.68%)	0.1844
Associated symptoms: Vomiting	448 (5.45%)	1,371 (3.23%)	<.0001
Associated symptoms: Cold sweat	2,420 (29.43%)	4,846 (11.4%)	<.0001
Associated symptoms: Dizziness	357 (4.34%)	3,053 (7.19%)	<.0001
Associated symptoms: Altered mental status	124 (1.51%)	776 (1.83%)	0.0455
Associated symptoms: Others	667 (8.11%)	4,327 (10.18%)	<.0001
Situation of onset			
Daily life	5,339 (64.94%)	29,541 (69.52%)	<.0001
Resting/sleeping	1,420 (17.27%)	6,442 (15.16%)	
Working	424 (5.16%)	1,388 (3.27%)	
Sports	296 (3.6%)	569 (1.34%)	
On education	4 (0.05%)	65 (0.15%)	
On transfer	312 (3.79%)	1,996 (4.7%)	
On Medical treatment	40 (0.49%)	309 (0.73%)	
Others	387 (4.71%)	2,181 (5.13%)	
Pain location: Left	2.430 (29.55%)	9,799 (23.06%)	<.0001
Pain location: Right	481 (5.85%)	2,020 (4.75%)	<.0001
Pain location: Substernal	1,678 (20.41%)	5,321 (12.52%)	<.0001
Pain location: Epigastric	2,637 (32.07%)	9,559 (22.5%)	<.0001
Pain location: Others	715 (8.7%)	2,379 (5.6%)	<.0001
Pain characteristics: pressure like	1,107 (13.46%)	4,193 (9.87%)	<.0001
Pain characteristics: tightening/squeezing	2,999 (36.48%)	9,596 (22.58%)	<.0001
Pain characteristics: bursting	356 (4.33%)	922 (2.17%)	<.0001

Pain characteristics: dissecting	478 (5.81%)	1,338 (3.15%)	<.0001
Pain characteristics: discomfort	2,081 (25.31%)	9,304 (22.13%)	<.0001
Pain characteristics: Others	1,009 (12.27%)	3,896 (9.17%)	<.0001
Pain scale (NRS)	5 ± 3.6	3.07 ± 3.45	<.0001
Radiating pain: left arm	991 (12.05%)	1,898 (4.47%)	<.0001
Radiating pain: right arm	412 (5.01%)	932 (2.19%)	<.0001
Radiating pain: back	695 (8.45%)	2,500 (5.88%)	<.0001
Radiating pain: neck	442 (5.38%)	1,386 (3.26%)	<.0001
Radiating pain: others	341 (4.15%)	1,269 (2.99%)	<.0001
Aggravating factor			
None	6,418 (85.93%)	24,443 (87.47%)	<.0001
Exercise	905 (12.12%)	2,750 (9.84%)	
Others	146 (1.95%)	752 (2.69%)	
Relieving factor			
None	5,450 (72.97%)	18,077 (64.69%)	<.0001
Nitroglycerin	1,086 (14.54%)	5,210 (18.64%)	
Resting	769 (10.3%)	3,854 (13.79%)	
Others	164 (2.2%)	804 (2.88%)	
Duration			
Under 5 min	767 (10.27%)	4,597 (16.45%)	<.0001
5 to 20 min	1,937 (25.93%)	7,566 (27.07%)	
Over 20 min	4,765 (63.8%)	15,782 (56.48%)	
Administration of medication	1,294 (16.76%)	7,032 (18.23%)	<.0001
Administration of nitroglycerin	1,548 (18.83%)	4,313 (10.15%)	<.0001
ECG monitoring device	7,820 (95.11%)	39,726 (93.49%)	<.0001
Electrode	6,978 (89.23%)	35,954 (90.5%)	<.0001
AED (automated external defibrillator)	842 (10.77%)	3,772 (9.5%)	
ECG rhythm findings			

Normal sinus rhythm	3,405 (43.54%)	21,701 (54.63%)	<.0001
Sinus tachycardia	536 (6.85%)	6,286 (15.82%)	
Sinus bradycardia	697 (8.91%)	1,933 (4.87%)	
Narrow QRS tachycardia	190 (2.43%)	1,689 (4.25%)	
Wide QRS tachycardia	152 (1.94%)	406 (1.02%)	
AV block	78 (1%)	271 (0.68%)	
Other AV blocks	29 (0.37%)	100 (0.25%)	
Unknown	729 (9.32%)	2,609 (6.57%)	
Others	2,004 (25.63%)	4,731 (11.91%)	
AV block			
Second degree AV block	237 (46.56%)	1,518 (48.41%)	0.0017
Third degree AV block	162 (31.83%)	967 (30.84%)	
Other AV block	110 (21.61%)	651 (20.76%)	
Wide QRS tachycardia			
Regular	112 (73.68%)	309 (76.11%)	<.0001
Irregular	40 (26.32%)	97 (23.89%)	
Narrow QRS tachycardia			
Regular	130 (68.42%)	1,163 (68.86%)	<.0001
Irregular	60 (31.58%)	526 (31.14%)	
ST segment elevation	2,426 (29.51%)	6,379 (15.01%)	<.0001
TIMI score	1.55 ± 1.2	1.15 ± 1.15	<.0001

Table S1(6): Baseline demographics for the test cohort of Dataset 2

**Variable**	**Testing set (N = 21,726)**		**P value**
	**I21.x (Yes = 3,560)**	**I21.x (No = 18,166)**	
Gender (Male)	2,819 (79.19%)	10,425 (57.39%)	<.0001
Age	63.17 ± 12.78	60.79 ± 17.24	<.0001
Location			
Seoul	922 (25.9%)	5,347 (29.43%)	<.0001
Others	2,638 (74.1%)	12,819 (70.57%)	
Time from symptom onset to ED arrival (min)	514.35 ± 11,928.72	319.01 ± 6,599.94	0.3427

Hypertension	1,373 (38.57%)	6,668 (36.71%)	0.0354
Diabetes mellitus	723 (20.31%)	3,199 (17.61%)	0.0001
Cerebrovascular disease	114 (3.2%)	735 (4.05%)	0.0175
Lung disease	48 (1.35%)	416 (2.29%)	0.0004
Cardiac disease	1,234 (34.66%)	9,034 (49.73%)	<.0001
Tuberculosis	16 (0.45%)	74 (0.41%)	0.7207
Hepatitis	7 (0.2%)	41 (0.23%)	0.7355
Liver cirrhosis	10 (0.28%)	76 (0.42%)	0.2323
Allergy	3 (0.08%)	16 (0.09%)	0.944
Cancer	65 (1.83%)	571 (3.14%)	<.0001
Renal disease	74 (2.08%)	493 (2.71%)	0.0297
Others	565 (15.87%)	3,920 (21.58%)	<.0001
Mental status			
A: alert	3,370 (94.66%)	17,324 (95.36%)	0.0002
P: react to pain	22 (0.62%)	208 (1.14%)	
U: unresponsive	38 (1.07%)	149 (0.82%)	
V: react to verbal stimulation	130 (3.65%)	485 (2.67%)	
Systolic blood pressure	80.64±21.17	82.58±19.69	<.0001
Diastolic blood pressure	127.89±32.06	132.7±30.09	<.0001
Heart rate	78.44±24.94	92.68±32.79	<.0001
Respiratory rate	18.77±5.32	18.72±5.16	0.6031
Body temperature	36.2±2.49	36.44±2.28	<.0001
Saturation	95.24±9.75	95.47±9.31	0.1985
Blood sugar	153.16±77.11	149.36±74.35	0.0068
Chief complaint: Chest pain	3,254 (91.4%)	11,850 (65.23%)	<.0001
Chief complaint: Dyspnea	996 (27.98%)	5,969 (32.86%)	<.0001
Chief complaint: Palpitation	51 (1.43%)	2,539 (13.98%)	<.0001
Chief complaint: Syncope	54 (1.52%)	1,606 (8.84%)	<.0001
Chief complaint: Others	59 (1.66%)	432 (2.38%)	0.0081
Associated symptoms: Nausea	183 (5.14%)	866 (4.77%)	0.3421
Associated symptoms: Vomiting	186 (5.22%)	573 (3.15%)	<.0001
Associated symptoms: Cold sweat	983 (27.61%)	2,062 (11.35%)	<.0001
Associated symptoms: Dizziness	151 (4.24%)	1,290 (7.1%)	<.0001

Associated symptoms: Altered mental status	57 (1.6%)	328 (1.81%)	0.3979
Associated symptoms: Others	270 (7.58%)	1,881 (10.35%)	<.0001
Situation of onset			
Daily life	2,401 (67.44%)	12,625 (69.5%)	<.0001
Resting/sleeping	562 (15.79%)	2,836 (15.61%)	
Working	168 (4.72%)	542 (2.98%)	
Sports	131 (3.68%)	249 (1.37%)	
On education	2 (0.06%)	30 (0.17%)	
On transfer	155 (4.35%)	833 (4.59%)	
On Medical treatment	14 (0.39%)	119 (0.66%)	
Others	127 (3.57%)	932 (5.13%)	
Pain location: Left	987 (27.72%)	4,147 (22.83%)	<.0001
Pain location: Right	187 (5.25%)	860 (4.73%)	0.1864
Pain location: Substernal	736 (20.67%)	2,244 (12.35%)	<.0001
Pain location: Epigastric	1,190 (33.43%)	4,087 (22.5%)	<.0001
Pain location: Others	299 (8.4%)	1,003 (5.52%)	<.0001
Pain characteristics: pressure like	488 (13.71%)	1,696 (9.34%)	<.0001
Pain characteristics: tightening/squeezing	1,295 (36.38%)	4,041 (22.24%)	<.0001
Pain characteristics: bursting	174 (4.89%)	412 (2.27%)	<.0001
Pain characteristics: dissecting	215 (6.04%)	528 (2.91%)	<.0001
Pain characteristics: discomfort	892 (25.06%)	4,018 (22.12%)	0.0001
Pain characteristics: Others	437 (12.28%)	1,709 (9.41%)	<.0001
Pain scale (NRS)	4.93±3.64	3.09±3.46	<.0001
Radiating pain: left arm	430 (12.08%)	812 (4.47%)	<.0001
Radiating pain: right arm	180 (5.06%)	339 (1.87%)	<.0001
Radiating pain: back	278 (7.81%)	1,092 (6.01%)	<.0001
Radiating pain: neck	191 (5.37%)	619 (3.41%)	<.0001
Radiating pain: others	152 (4.27%)	545 (3%)	<.0001
Aggravating factor			
None	2,809 (86.32%)	10,348 (87.32%)	<.0001
Exercise	377 (11.59%)	1,125 (9.49%)	
Others	68 (2.09%)	377 (3.18%)	
Relieving factor			

None	2,381 (73.17%)	7,641 (64.48%)	<.0001
Nitroglycerin	464 (14.26%)	2,137 (18.03%)	
Resting	340 (10.45%)	1,730 (14.6%)	
Others	69 (2.12%)	342 (2.89%)	
Duration			
Under 5 min	353 (10.85%)	1,936 (16.34%)	<.0001
5 to 20 min	821 (25.23%)	3,285 (27.72%)	
Over 20 min	2,080 (63.92%)	6,629 (55.94%)	
Administration of medication	538 (16.26%)	2,925 (17.81%)	<.0001
Administration of nitroglycerin	655 (18.4%)	1,847 (10.17%)	<.0001
ECG monitoring device	3,387 (95.14%)	17,017 (93.67%)	0.0008
Electrode	2,995 (88.43%)	15,355 (90.23%)	<.0001
AED (automated external defibrillator)	392 (11.57%)	1,662 (9.77%)	
ECG rhythm findings			
Normal sinus rhythm	1,546 (45.65%)	9,259 (54.41%)	<.0001
Sinus tachycardia	208 (6.14%)	2,689 (15.8%)	
Sinus bradycardia	333 (9.83%)	823 (4.84%)	
Narrow QRS tachycardia	75 (2.21%)	730 (4.29%)	
Wide QRS tachycardia	52 (1.54%)	207 (1.22%)	
AV block	34 (1%)	109 (0.64%)	
Other AV blocks	9 (0.27%)	41 (0.24%)	
Unknown	304 (8.98%)	1,149 (6.75%)	
Others	826 (24.39%)	2,010 (11.81%)	
AV block			
Second degree AV block	96 (44.44%)	641 (49.35%)	0.0579
Third degree AV block	67 (31.02%)	391 (30.1%)	
Other AV block	53 (24.54%)	267 (20.55%)	
Wide QRS tachycardia			
Regular	43 (82.69%)	161 (77.78%)	0.1911
Irregular	9 (17.31%)	46 (22.22%)	

Narrow QRS tachycardia			
Regular	43 (57.33%)	519 (71.1%)	<.0001
Irregular	32 (42.67%)	211 (28.9%)	
ST segment elevation	1,053 (29.58%)	2,723 (14.99%)	<.0001
TIMI score	1.59 ± 1.2	1.15 ± 1.15	<.0001

Table S2: Odds ratios for event

Table S2(1). Odds ratios for event of Dataset 1, A-type model

**Variable**	**Odds ratio**	**95% CI**	**P value**
Gender (Male)	2.362	2.243, 2.488	<.0001
Age	1.031	1.029, 1.033	<.0001
Location			
Seoul	-	-	-
Others	1.119	1.064, 1.177	<.0001
Time from symptom onset to emergency department (ED) arrival (min)	1	1, 1	0.2046
Hypertension	1.032	0.982, 1.084	0.2149
Diabetes mellitus	1.241	1.171, 1.315	<.0001
Cerebrovascular disease	0.829	0.745, 0.922	0.0006
Lung disease	0.395	0.351, 0.444	<.0001
Cardiac disease	0.578	0.549, 0.609	<.0001
Tuberculosis	0.638	0.456, 0.891	0.0084
Hepatitis	1.016	0.645, 1.598	0.9464
Liver cirrhosis	0.448	0.303, 0.662	<.0001
Allergy	0.261	0.103, 0.658	0.0044
Cancer	0.57	0.5, 0.65	<.0001
Renal disease	0.916	0.802, 1.047	0.1988
Others	0.693	0.653, 0.736	<.0001
Mental status			
A: alert			
P: react to pain	0.819	0.678, 0.988	0.037
U: unresponsive	1.046	0.824, 1.326	0.7132
V: react to verbal stimulation	1.145	1.015, 1.292	0.0278
Systolic blood pressure	1.005	1.003, 1.007	<.0001
Diastolic blood pressure	0.994	0.992, 0.995	<.0001
Heart rate	0.991	0.99, 0.992	<.0001

Respiratory rate	1.015	1.011, 1.019	<.0001
Body temperature	1.008	0.997, 1.019	0.157
Saturation	0.994	0.991, 0.997	<.0001
Blood sugar	1.001	1.001, 1.001	<.0001
Chief complaint: Chest pain	2.311	1.959, 2.726	<.0001
Chief complaint: Dyspnea	0.889	0.837, 0.945	0.0002
Chief complaint: Palpitation	0.397	0.327, 0.482	<.0001
Chief complaint: Syncope	0.246	0.211, 0.286	<.0001
Chief complaint: Others	1.629	1.357, 1.956	<.0001
Associated symptoms: Nausea	1.01	0.899, 1.135	0.8644
Associated symptoms: Vomiting	1.422	1.266, 1.596	<.0001
Associated symptoms: Cold sweat	2.609	2.461, 2.766	<.0001
Associated symptoms: Dizziness	0.699	0.622, 0.785	<.0001
Associated symptoms: Altered mental status	1.078	0.908, 1.279	0.3909
Associated symptoms: Others	0.851	0.785, 0.922	<.0001
Situation of onset			
Daily life	-	-	-
Resting/sleeping	1.031	0.97, 1.097	0.3242
Working	1.661	1.471, 1.877	<.0001
Sports	3.118	2.654, 3.662	<.0001
On education	0.411	0.143, 1.185	0.0999
On transfer	1.045	0.925, 1.181	0.4762
On Medical treatment	0.709	0.539, 0.935	0.0146
Others	0.879	0.788, 0.981	0.0216
Pain location: Left	1.1	0.987, 1.225	0.0847
Pain location: Right	0.781	0.696, 0.877	<.0001
Pain location: Substernal	1.342	1.199, 1.502	<.0001
Pain location: Epigastric	1.233	1.105, 1.375	0.0002
Pain location: Others	1.375	1.205, 1.569	<.0001
Pain characteristics: pressure like	1.226	1.1, 1.367	0.0002
Pain characteristics: tightening/squeezing	1.505	1.359, 1.667	<.0001
Pain characteristics: bursting	1.518	1.305, 1.766	<.0001
Pain characteristics: dissecting	1.528	1.33, 1.755	<.0001
Pain characteristics: discomfort	0.95	0.858, 1.05	0.3144
Pain characteristics: Others	1.118	0.994, 1.257	0.0624

Pain scale (NRS)	1.063	1.054, 1.071	<.0001
Radiating pain: left arm	2.251	2.042, 2.482	<.0001
Radiating pain: right arm	1.109	0.962, 1.279	0.154
Radiating pain: back	0.928	0.844, 1.02	0.1193
Radiating pain: neck	1.133	1.005, 1.277	0.0404
Radiating pain: others	1.021	0.899, 1.16	0.7441
Aggravating factor			
None	-	-	-
Exercise	1.215	1.111, 1.328	<.0001
Others	0.821	0.687, 0.981	0.03
Relieving factor			
None	-	-	-
Nitroglycerin	0.749	0.692, 0.811	<.0001
Resting	0.629	0.577, 0.687	<.0001
Others	0.695	0.583, 0.828	<.0001
Duration			
Under 5 min	-	-	-
5 to 20 min	1.244	1.137, 1.361	<.0001
Over 20 min	1.427	1.315, 1.548	<.0001

Table S2(2). Odds ratios for event of Dataset 2, A-type model

**Variable**	**Odds ratio**	**95% CI**	**P value**
Gender (Male)	2.598	2.438, 2.768	<.0001
Age	1.027	1.025, 1.029	<.0001
Location			
Seoul	-	-	-
Others	1.101	1.037, 1.17	0.0017
Time from symptom onset to emergency department (ED) arrival (min)	1	1, 1	0.0108
Hypertension	0.969	0.914, 1.027	0.2849
Diabetes mellitus	1.184	1.104, 1.27	<.0001
Cerebrovascular disease	0.951	0.832, 1.086	0.4568
Lung disease	0.563	0.453, 0.7	<.0001
Cardiac disease	0.419	0.395, 0.444	<.0001
Tuberculosis	0.585	0.402, 0.85	0.005
Hepatitis	0.552	0.277, 1.099	0.0908
Liver cirrhosis	0.338	0.188, 0.607	0.0003

Allergy	0.79	0.271, 2.3	0.6657
Cancer	0.735	0.615, 0.88	0.0008
Renal disease	0.858	0.716, 1.027	0.0957
Others	0.765	0.711, 0.823	<.0001
Mental status			
A: alert			
P: react to pain	1.068	0.789, 1.444	0.6706
U: unresponsive	0.791	0.566, 1.106	0.1703
V: react to verbal stimulation	1.462	1.234, 1.732	<.0001
Systolic blood pressure	1.005	1.003, 1.007	<.0001
Diastolic blood pressure	0.994	0.992, 0.995	<.0001
Heart rate	0.989	0.988, 0.99	<.0001
Respiratory rate	1.02	1.014, 1.025	<.0001
Body temperature	1.016	1.001, 1.03	0.0322
Saturation	0.995	0.991, 0.999	0.0227
Blood sugar	1.001	1.001, 1.001	<.0001
Chief complaint: Chest pain	1.379	1.135, 1.675	0.0012
Chief complaint: Dyspnea	0.992	0.929, 1.059	0.8099
Chief complaint: Palpitation	0.311	0.253, 0.384	<.0001
Chief complaint: Syncope	0.251	0.205, 0.308	<.0001
Chief complaint: Others	1.207	0.98, 1.487	0.0767
Associated symptoms: Nausea	0.942	0.826, 1.074	0.3732
Associated symptoms: Vomiting	1.592	1.393, 1.82	<.0001
Associated symptoms: Cold sweat	2.367	2.219, 2.526	<.0001
Associated symptoms: Dizziness	0.678	0.597, 0.77	<.0001
Associated symptoms: Altered mental status	0.889	0.704 1.122	0.3204
Associated symptoms: Others	0.88	0.799, 0.969	0.0092
Situation of onset			
Daily life	-	-	-
Resting/sleeping	1.003	0.934, 1.077	0.9399
Working	1.581	1.385, 1.803	<.0001
Sports	2.8	2.347, 3.339	<.0001
On education	0.463	0.151, 1.416	0.1769
On transfer	0.942	0.821, 1.082	0.399
On Medical treatment	0.755	0.526, 1.083	0.1267

Others	0.993	0.875, 1.126	0.9084
Pain location: Left	1.067	0.949, 1.198	0.2774
Pain location: Right	0.838	0.74, 0.949	0.0055
Pain location: Substernal	1.339	1.186, 1.512	<.0001
Pain location: Epigastric	1.211	1.076, 1.361	0.0014
Pain location: Others	1.312	1.136, 1.515	0.0002
Pain characteristics: pressure like	1.096	0.977, 1.23	0.1166
Pain characteristics: tightening/squeezing	1.315	1.18, 1.465	<.0001
Pain characteristics: bursting	1.348	1.148, 1.583	0.0003
Pain characteristics: dissecting	1.302	1.123, 1.51	0.0005
Pain characteristics: discomfort	0.933	0.839, 1.037	0.1974
Pain characteristics: Others	1.106	0.976, 1.254	0.1145
Pain scale (NRS)	1.06	1.051, 1.07	<.0001
Radiating pain: left arm	2.016	1.822, 2.231	<.0001
Radiating pain: right arm	1.064	0.918, 1.233	0.4078
Radiating pain: back	0.917	0.831, 1.012	0.0864
Radiating pain: neck	1.091	0.964, 1.236	0.1673
Radiating pain: others	0.975	0.852, 1.116	0.7136
Aggravating factor			
None	-	-	-
Exercise	1.257	1.146, 1.379	<.0001
Others	0.876	0.718, 1.068	0.1902
Relieving factor			
None	-	-	-
Nitroglycerin	0.721	0.665, 0.781	<.0001
Resting	0.581	0.529, 0.639	<.0001
Others	0.696	0.575, 0.841	0.0002
Duration			
Under 5 min	-	-	-
5 to 20 min	1.319	1.195, 1.455	<.0001
Over 20 min	1.555	1.421, 1.701	<.0001

Table S2(3). Odds ratios for event of Dataset 2, B-type model

**Variable**	**Odds ratio**	**95% CI**	**P value**
Gender (Male)	2.419	2.267, 2.58	<.0001
Age	1.018	1.015, 1.02	<.0001

Location			
Seoul	-	-	-
Others	1.013	0.951, 1.078	0.6929
Time from symptom onset to emergency department (ED) arrival (min)	1	1, 1	0.0041
Hypertension	0.951	0.896, 1.01	0.1
Diabetes mellitus	1.116	1.038, 1.199	0.0028
Cerebrovascular disease	0.911	0.796, 1.044	0.1792
Lung disease	0.548	0.439, 0.683	<.0001
Cardiac disease	0.356	0.334, 0.38	<.0001
Tuberculosis	0.511	0.349, 0.747	0.0005
Hepatitis	0.541	0.271, 1.081	0.082
Liver cirrhosis	0.356	0.197, 0.644	0.0006
Allergy	0.669	0.221, 2.022	0.4757
Cancer	0.77	0.642, 0.924	0.005
Renal disease	0.814	0.678, 0.977	0.0269
Others	0.749	0.695, 0.807	<.0001
Mental status			
A: alert	-	-	-
P: react to pain	0.995	0.73, 1.358	0.9772
U: unresponsive	0.656	0.464, 0.926	0.0164
V: react to verbal stimulation	1.434	1.206, 1.705	<.0001
Systolic blood pressure	1.005	1.003, 1.007	<.0001
Diastolic blood pressure	0.994	0.993, 0.996	<.0001
Heart rate	0.99	0.988, 0.991	<.0001
Respiratory rate	1.018	1.012, 1.023	<.0001
Body temperature	1.014	0.999, 1.029	0.0666
Saturation	0.997	0.993, 1.001	0.1044
Blood sugar	1.001	1.001, 1.001	<.0001
Chief complaint: Chest pain	1.443	1.184, 1.759	0.0003
Chief complaint: Dyspnea	0.943	0.881, 1.008	0.0847
Chief complaint: Palpitation	0.298	0.242, 0.368	<.0001
Chief complaint: Syncope	0.255	0.208, 0.314	<.0001
Chief complaint: Others	0.999	0.808, 1.235	0.9936
Associated symptoms: Nausea	0.92	0.805, 1.052	0.2255
Associated symptoms: Vomiting	1.535	1.339, 1.76	<.0001
Associated symptoms: Cold sweat	2.196	2.055, 2.346	<.0001

Associated symptoms: Dizziness	0.669	0.588, 0.762	<.0001
Associated symptoms: Altered mental status	0.884	0.698, 1.12	0.3071
Associated symptoms: Others	0.85	0.771, 0.938	0.0012
Situation of onset			
Daily life	-	-	-
Resting/sleeping	1.009	0.938, 1.085	0.8081
Working	1.542	1.347, 1.766	<.0001
Sports	2.659	2.218, 3.188	<.0001
On education	0.319	0.102, 1.003	0.0507
On transfer	0.961	0.836, 1.106	0.5836
On Medical treatment	0.736	0.512, 1.059	0.0989
Others	1.019	0.897, 1.159	0.7679
Pain location: Left	1.045	0.927, 1.177	0.4713
Pain location: Right	0.835	0.735, 0.948	0.0054
Pain location: Substernal	1.298	1.146, 1.47	<.0001
Pain location: Epigastric	1.178	1.045, 1.329	0.0075
Pain location: Others	1.277	1.102, 1.479	0.0011
Pain characteristics: pressure like	1.063	0.945, 1.196	0.3068
Pain characteristics: tightening/squeezing	1.254	1.122, 1.4	<.0001
Pain characteristics: bursting	1.296	1.1, 1.528	0.002
Pain characteristics: dissecting	1.255	1.079, 1.461	0.0033
Pain characteristics: discomfort	0.905	0.812, 1.008	0.0706
Pain characteristics: Others	1.073	0.944, 1.219	0.2823
Pain scale (NRS)	1.046	1.037, 1.056	<.0001
Radiating pain: left arm	1.932	1.742, 2.142	<.0001
Radiating pain: right arm	1.061	0.913, 1.233	0.4378
Radiating pain: back	0.916	0.828, 1.013	0.0874
Radiating pain: neck	1.08	0.952, 1.226	0.2322
Radiating pain: others	0.949	0.827, 1.09	0.4603
Aggravating factor			
None	-	-	-
Exercise	1.159	1.055, 1.275	0.0022
Others	0.844	0.689, 1.033	0.1003
Relieving factor			
None	-	-	-

Nitroglycerin	0.673	0.611, 0.741	<.0001
Resting	0.627	0.57, 0.689	<.0001
Others	0.724	0.597, 0.878	0.001
Duration			
Under 5 min	-	-	-
5 to 20 min	1.279	1.157, 1.413	<.0001
Over 20 min	1.409	1.285, 1.544	<.0001
Administration of medication	0.979	0.898, 1.068	0.6372
Administration of nitroglycerin	1.301	1.199, 1.412	<.0001
ECG monitoring device	0.803	0.68, 0.949	0.0099
Electrode	-	-	-
AED (automated external defibrillator)	1.04	0.949, 1.14	0.3994
ECG rhythm findings			
Normal sinus rhythm	-	-	-
Sinus tachycardia	1.114	0.993, 1.25	0.0647
Sinus bradycardia	1.558	1.388, 1.748	<.0001
Narrow QRS tachycardia	1.462	1.178, 1.813	0.0006
Wide QRS tachycardia	3.037	2.344, 3.935	<.0001
Atrioventricular (AV) block	1.437	1.052, 1.962	0.0227
Other AV blocks	1.663	1.03, 2.685	0.0376
Unknown	1.628	1.472, 1.799	<.0001
Others	2.162	2.004, 2.332	<.0001
AV block			
Second degree AV block	-	-	-
Third degree AV block	0.893	0.7, 1.139	0.3627
Other AV block	1.044	0.794, 1.373	0.7593
Wide QRS tachycardia			
Regular	-	-	-
Irregular	1.008	0.62, 1.64	0.9746
Narrow QRS tachycardia			
Regular	-	-	-
Irregular	0.856	0.6, 1.222	0.3926
ST segment elevation	1.369	1.28, 1.463	<.0001
Thrombolysis in myocardial infarction (TIMI) score	1.225	1.19, 1.262	<.0001

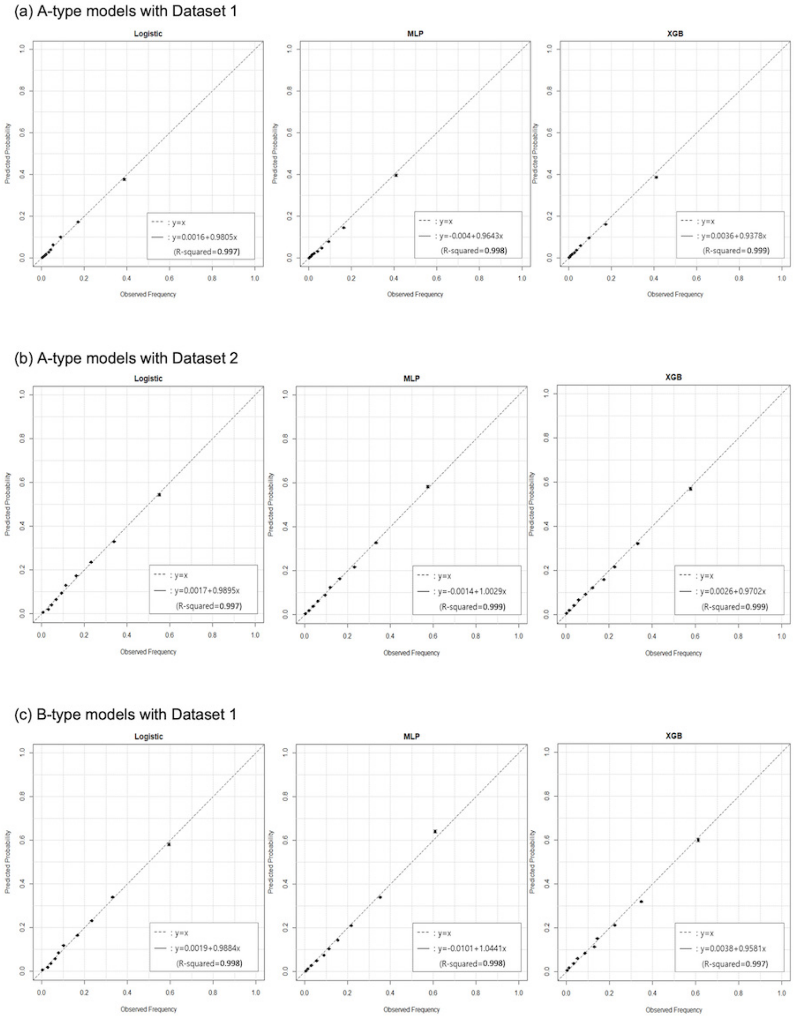

Figure S1: the calibration plot for all the tested models
